# Extracardiac Fontan with T-shape conduit in non-confluent pulmonary arteries

**DOI:** 10.1186/1749-8090-3-7

**Published:** 2008-02-20

**Authors:** Jae Gun Kwak, Jeong Ryul Lee, Woo-Sung Jang, Eun Jung Bae

**Affiliations:** 1 Department of Thoracic and Cardiovascular Surgery, Seoul National University Children's Hospital, Seoul National University College of Medicine, Seoul National University Clinical Research Institute, Seoul, South Korea; 2 Department of Pediatrics, Seoul National University Children's Hospital, Seoul National University College of Medicine, Seoul, South Korea

## Abstract

A 34 months-old male patient with double inlet right ventricle with nonconfluent pulmonary arteries who underwent successful extracardiac fenestarated Fontan procedure using pre-designed T-shaped PTFE vascular graft after multi-step rehabilitation of the diminutive hilar pulmonary arteries. At first we performed 6 mm confluent pulmonary artery vascular graft implantation with 4 mm BT shunt at patient's 4 weeks old. At 9 months of patient, we upsized the confluent pulmonary arterial graft to 8 mm with bidirectional cavopulmonary connection, and, at 34 months, we performed extracardiac conduit Fontan procedure with pre-designed T-shape conduit including the confluent pulmonary arterial portion at last. Patient shows excellent functional status and development.

## Introduction

Intrapericardial pulmonary artery creation is inevitable at neonatal period because patient had no confluent pulmonary artery and compliance of pulmonary vascular bed is not ready to accept pulmonary boold flow. Furthermore, adequate inflow pulmonary bed preparation for systemic venous return is crucial for definitive Fontan repair. In this situation, we thought multi-step approach would be helpful for the pulmonary bed growth.

## Case description

A 4 week-old male diagnosed with {S, X, D} type double inlet right ventricle, small left side atrioventricular valve, intact atrial septum, and non-confluent pulmonary arteries with diminutive hilar pulmonary arteries (2.7 and 3 mm diameter) supplied by bilateral patent ductuses was admitted to our unit. Because disconnected pulmonary arterial portion was too long and pulmonary artery hilar portion was too tiny to do unifocalization, we performed central pulmonary artery interposition between both pulmonary arterial hilar portion with 6 mm polytetrafluoroethylene(PTFE) vascular graft and left modified Blalock-Taussig shunt with 4 mm PTFE graft and ductus division under median full sternotomy. Postoperative arterial saturation was 85%.

**Figure 1:**
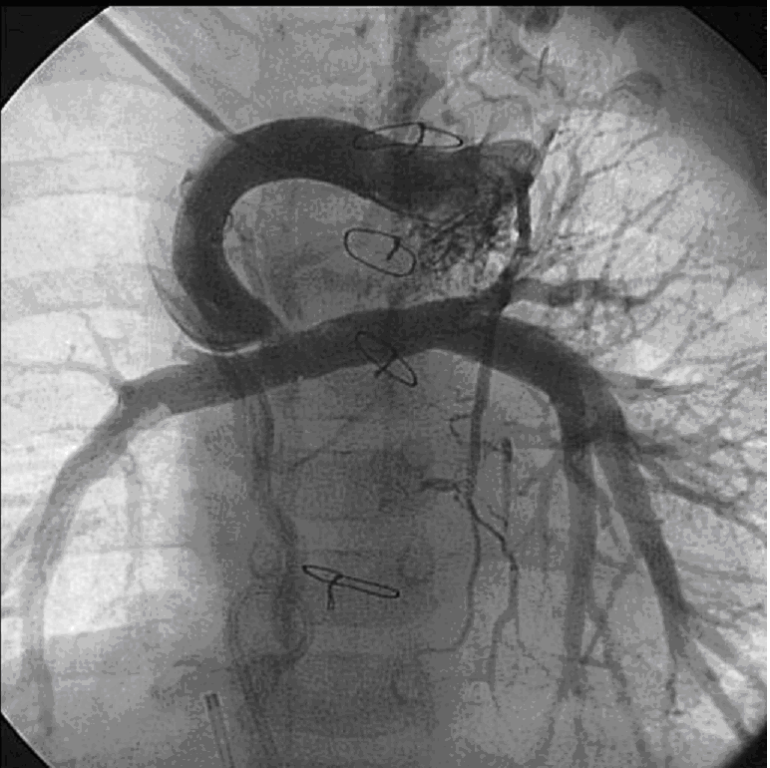
**Preoperative angiographic finding.** Focal stenosis between bilateral cavo-pulmonary shunt and pulmonary artery, numerous collaterals arised from both internal mammary arteries.

At 9 months, echocardiography and cardiac catheterizing showed that previous left modified BT shunt was stenotic at the anastomosis site with artificial confluent pulmonary artery (PTFE 6 mm vascular graft), and left pulmonary artery orifice was totally occluded, it's distal part was filled by collateral vessels. Both pulmonary arteries were relatively small to his weight and age. We changed 6 mm central pulmonary artery conduit wiht upsized 8 mm PTFE graft and right bidirectional cavopulmonary connection and atrial septectomy was performed concomitantly. During the interstage period after above 2 staged palliative operations, we used coumadin for anticoagulation, tried to maintain INR level between 1.5 and 2.0. At 32 months (14 kg), second cardiac catheterization revealed pressure gradient of 11 mmHg at anastomosis site between SVC (19 mmHg) and central pulmonary arterial conduit (8 mmHg), and multiple collaterals from both internal mammary arteries (fig. 1). Pulmonary resistance was 1.2 wood unit. Collateral vessels from right side internal mammary artery were coil-embolized during catheterization. We already planed to do Fontan procedure sooner or later, did not decide to do catheterized based intervention for the stenotic site between SVC and artificial pulmonary artery. In addition, we thought it would be dangerous to do interventional access for that stenotic site due to intimal calcified or fibrotic peel formation within the PTFE conduit. At second operation, we confirmed intimal fibrotic peel formation in the PTFE conduit in the operating room.

**Figure 2:**
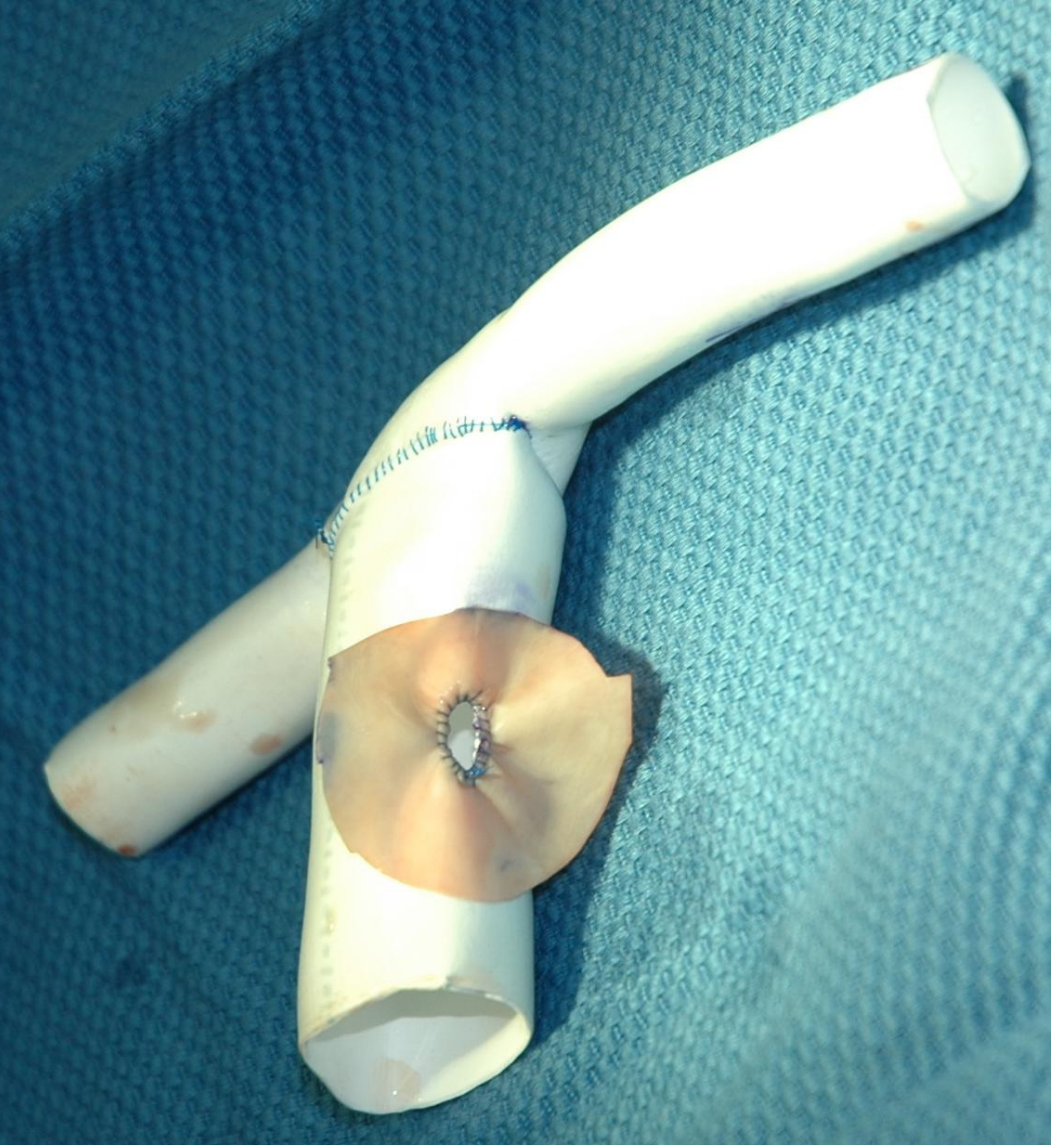
Pre-designed T-shaped Gore-Tex graft with 4.5 mm bovine fenestration.

**Figure 3:**
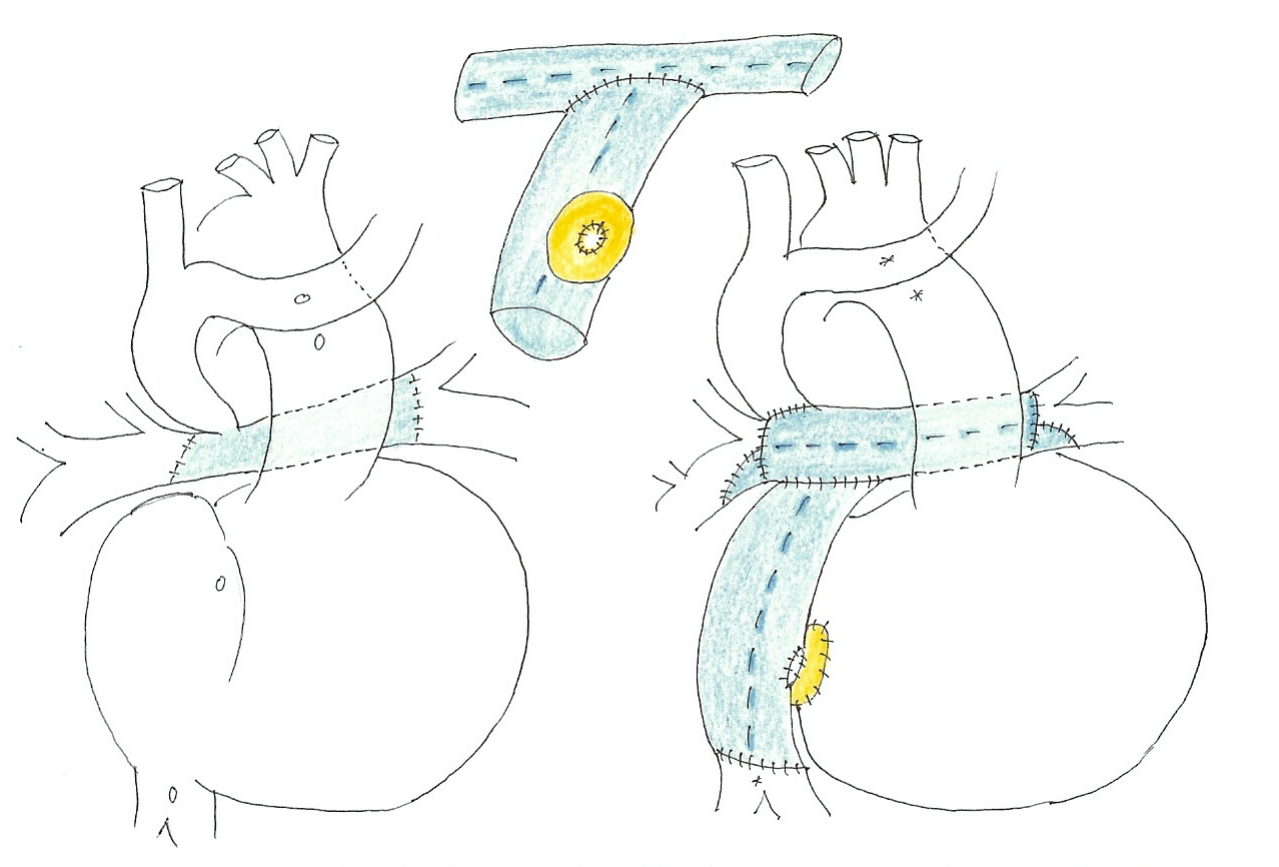
**Schematic operative findings.** (left ; preoperative state, center ; T-shaped Fontan conduit, right ; postoperative state).

At 34 months(14.5 kg), extracardiac conduit Fontan operation was performed, replacing central pulmonary arterial tube graft with predesigned T-shaped conduit which was made of 12 mm and 20 mm sized PTFE grafts including 4.5 mm fenestration made of bovine pericardial skirt (fig. 2). Hilar pulmonary arteries were enlarged using PTFE graft patch to overcome size discrepancy between native pulmonary arteries (hilar portion) and both arms of T-shape conduit. (fig. 3)

Under the median sternotomy and moderate hypothermia, standard technique for cardiopulmonary bypass was used with aortic and direct superior and inferior caval venous cannulation. The atrium was vented. We transected IVC and closed cardiac end of IVC with 5-0 prolene. IVC-extracardiac conduit anastomosis was done using 5-0 prolene following removal of previous confluent pulmonary artery conduit. Then both pulmonary artery hilums were enlarged with PTFE vascular graft using 6-0 prolene. Both pulmonary arteries were anastomosed with 12 mm PTFE vascular graft with 5-0 prolene, and SVC was connected with 6-0 prolene after creation of large opening. The heart was arrested for anastomosis of prepared fenestration site and right atrial wall. Patient was weaned from cardiopulmonary bypass smoothly. Total cardiopulmonary bypass time was 266 minutes and aortic clamping time was 21 minutes. An intraoperative transesophageal echocardiography showed wide and patent Fontan pathway with an adequate flow through fenestration.

Postoperative mean CVP was 16 mmHg and maintained with this level. Electrocardiography showed normal sinus rhythm. Extubation was done postoperative day 3 and hospital course was smooth. One year after operation, echocardiography showed good Fontan pathway, confluent pulmonary artery and excellent ventricular function without evidence of thromboembolism. The patient was been on Coumadin for prothrombin time INR 2.

## Discussion

In this particular case, intrapericardial pulmonary artery creation was inevitable at neonatal period because the patient had no confluent pulmonary artery and pulmonary vascular bed was not good. Furthermore, adequate inflow bed preparation for the systemic venous return is crucial for definitive Fontan repair.

Fortunately, with central pulmonary artery (6 mm) and BT shunt (4 mm), pulmonary artery hilar area was well-grown for bidirectional cavo-pulmonary connection, at the 2^nd ^statge operation. Considering patient's age and body weight for the future Fontan procedure, central pulmonary arterial conduit was replaced with 8 mm PTFE graft at the time of BCPS.

Cardiac catheterization demonstrated 11 mmHg of pressure gradient between SVC and RPA and 92% of oxygen saturation at 32 months. Apparent facial edema lead to suspect SVC obstruction. From this, we decided to proceed the Fontan procedure with re-replacement of central pulmonary artery conduit. It is well known that pulmonary atresia with non-confluent pulmonary artery is risk factor for Fontan operation. In this condition, size of both pulmonary hilar and pulmonary artery resistance were very important for successful Fontan procedure [1]. Although central pulmonary artery tube is enough and adequate for Fontan pathway now, repeated upsizing of pulmonary conduit may be required until it reaches the adult size. In this regards, continued close follow up is mandatory.

Pre-building T-shaped conduit composed of 12 mm PTFE tube for central pulmonary artery and 20 mm PTFE tube for extracardiac conduit outside the operation table was very helpful. This procedure shortened bypass time as well as the facilitated the anastomosis. Considering future growth-mismatch, enlargement of native hilar pulmonary arteries as large as possible was required to fit the end of 12 mm tube.

Even though several options were reported graft material including pericardial roll, xenopericardium, etc, we used PTFE vascular graft, because pericardial conduit, especially, heterologous pericardial roll has several problem like calcification, thrombosis and bacterial infection [2-4]. Having changed the up-sized confluent pulmonary conduit at adequate timing repeatedly helped to gain well-balanced both pulmonary arteries' growth without increasing pulmonary artery resistance (<1.2 wood unit). Considering body surface area, 12 mm pulmonary artery conduit, that patient has now, can be used till the body surface area become 2.0 m^2^. Because the Z-value of left and right pulmonary arteries are -3 and -2 respectively with this conduit size at body surface area, so if there will not occur the stenosis in the conduit, we could use this conduit for a long time without conduit change. For extracardiac conduit, patient has 20 mm sized conduit now, this is about 85% of adult average IVC size, 24 mm. Also, if the stenotic complication may not occured, we could use this extracardiac conduit without change[5,6]. Untill now, patient showes the excellent hemodynamics and functional status.

## Conclusion

We report a 34 months-old male patient with double inlet right ventricle associated with nonconfluent pulmonary arteries who underwent successful extracardiac fenestarated Fontan procedure using pre-designed T-shaped PTFE vascular graft after multi-step rehabilitation of the diminutive hilar pulmonary arteries.
